# Yellow Fever Antidote

**Published:** 1858-02

**Authors:** 


### YELLOW FEVER ANTIDOTE.

A PORTUGUESE Medical Journal states, that when the Yellow Fever prevailed at *Lisbon*, all those persons escaped who lived in houses lighted by gas.

We have several times stated in these pages, that those who lived where fever and ague and chills and fever prevailed, might avoid them by the practicable expedient of kindling a brisk fire in the family-room at daylight and at sundown throughout the year (and eating breakfast before going out to work): the philosophy of the fact, in both cases, being the same, that heat, from whatever source, rarefies the impregnated air, making it, in the first place, less concentrated, and, therefore, less malignant, besides causing it to ascend above the breathing-point, and producing those drafts of air, which, under all circumstances, are self-purifying. The antagonistic properties of fire against all the fevers of this country, from the simple chill and fever, up to the deadly "congestive," and the malignant "yellow," we have many a time verified in our own person, in places where these diseases love to revel.

